# St. Thomas's Hospital Reports

**Published:** 1837-01

**Authors:** 


### Art. VIII.

1. *St. Thomas's Hospital Reports.* No. III. and IV. *April and June*, 1836.
2. *Guy's Hospital Reports.* No. III. *September*, 1836.

The medical officers of Guy's and St. Thomas's Hospital continue to support with vigour the publication of the Reports from their respective hospitals. The two Numbers of the St. Thomas's Reports now before us are fully equal to their predecessors. The third Number of the Reports from Guy's Hospital shows a great improvement in the manner in which it is illustrated. It is really a very handsome book, and contains no less than twelve plates. Most of these are engraved on zinc by Mr. Canton, draughtsman to the Hospital, and they unite the distinctness of copper-plate engravings with the soft chalkiness of lithographs. The minute anatomy of malignant tubera of the liver (p. 651,) is an admirable specimen. Some are coloured, and very naturally; the plates of iritis and hypopium, illustrating Mr. Morgan's cases, have all that dull yet definite indistinctness of colouring seen in the original disorganization. It would be difficult to form any just estimate of the comparative merits of the contents of the two rival publications; in both, as might be expected, with some extremely good papers, there are some indifferent ones. The volume from St. Thomas's is, as its name imports, exclusively a report of cases, with clinical observations; the other takes a wider range, and is not confined to information furnished by the wards of its Hospital only.

The first case of the third Number of the St. Thomas's Hospital Reports is one of *Porrigo Lupinosa of the Head.* It is reported by Mr. Clark, and makes a text to Dr. Roots for some valuable practical observations on the treatment of that troublesome disease. Dr. Roots justly insists that there is no *one plan*, but that the disease must be treated according to its existing condition. Generally speaking, the tar-ointment is the most useful application; but, if there is much excitement in the skin, it is too stimulating; and Ceratum plumbi acet. or Ung. zinci may be employed. If there is much inflammation, the best applications are rags dipped in water, or cold solutions of the salts of lead, or even leeches. If tar-ointment is not sufficiently stimulating, it may be combined with Ung. hydr.-nitratis, or with the Ung. sulphuris comp. The plan requires frequently to be changed in the course of the disease; and, when a sufficient stimulus has been given, the milder ointments substituted until the excitement has subsided. Generally, when it is thought necessary to produce excitement, this should be done gradually; for example, one part of Ung. picis either with two or three parts of Ung. zinci may be applied, and then the latter may be gradually diminished until the Ung. picis is used pure. Most stimulants have occasionally succeeded and failed, chiefly from inattention to the peculiar circumstances of the case, and want of cleanliness. In conjunction with local applications, the most efficient means is removal of the hair; for the eruption never exists long without the bulbs becoming diseased, and, as long as these continue affected, unless they are removed, a fresh crop of pustules will continue to spring up. The best way is to remove each hair with a pair of tweezers; and it is impossible to do this properly without a lens. When these applications fail, Dr. Roots uses the pyroligneous acid, which he has often found of great advantage, or creosote. No constitutional treatment is required unless the general health is impaired. If there is a cachectic state of the system, good generous diet should be allowed, and perhaps tonic medicines: a plethoric state demands contrary means. Generally speaking, a linen cap is preferable to oiled silk, which keeps the part too excited; where there is a want of activity in the vessels of the part, the application of the oiled-silk cap for two or three days may be useful, but otherwise, says Dr. Roots, "I am quite sure you will not find any permanent benefit from its continued application."

The next case is one of considerable interest, and gives Dr. Roots an opportunity of furnishing some excellent remarks on the subject of Hysteria. The case is headed "*Hysterical Paralysis.*" A young unmarried woman, æt. twenty-four, was admitted with paralysis of the right arm and leg, of about a week's standing. Twelve days before, she had suffered great pain in the head, which lasted until the sudden attack of hemiplegia. She was low spirited, and complained of a frequent sensation of choking; the pain in the head continued, and there was pain in the epigastrium. Leeches were applied in large quantities to the præcordia, and five grains of Hydr. cum Cretâ were given twice daily, and persevered in for a fortnight. The motion returned in her right arm, and the pulse became more rapid, the other symptoms continued the same. At this time Dr. Roots (who had been out of town) saw her, and observing that the pulse was more frequent, that she was pallid, complained of choking, and occasionally fainted, he ordered an ounce and a half of infusion of valerian, one drachm of aromatic spirit of ammonia, and half a drachm of tincture of hyoscyamus, three times a day, with a scruple of carbonate of soda. This was followed with great improvement; but, finding that the menstruation was irregular, and looking at the origin of the disease as congestion about the uterus, Dr. Roots ordered ten ounces of blood to be taken from the sacrum by cupping. Her diet was improved, she was allowed porter, and subsequently two grains of quinine, two grains of sulphate of iron, and half a drachm of tincture of hyoscyamus, three times a day. Her general health improved, and she gradually regained the use of her leg. It was curious to observe how strong the influence of the mind was in aiding her recovery. "For a length of time, she could only move her toes and gradually draw up the leg: she seemed afraid, she had persuaded herself that she was perfectly incapable of doing it; but, on insisting that she should quit her bed, and attempt to stand, telling her she should be supported by people in the ward, she found that she really was capable of doing more than she imagined. Still there was that degree of hesitation, that degree of trepidation, in attempting to use the limb, which made it plain that it was the result, to a considerable extent, of fear." (P. 254.) To work on her mind, Dr. R. requested the clerk to mention in her hearing that a large blister should be applied in two days, were there no amendment. By means of the strong impression thus produced, she day by day improved without the necessity of applying it at all.

With regard to the treatment of hysteria generally, Dr. Roots strongly advises bleeding from the arm if there is much general excitement, and local depletion if there is local excitement. Where it is connected with any thing like congestion about the uterus, cupping the loins or sacral region, or leeching the vulva; when in conjunction with this there is pain in the occiput, he also cups that part, or even bleeds from the arm. Purgatives are always of use; one of the best is from three to five drachms of oil of turpentine with half an ounce of castor oil, which is "a warm stimulating purgative, which empties the bowels thoroughly, and, by its stimulating effect on the muscular coat of the intestines, exercises a degree of tonic power over them, and prevents that enormous distention which so frequently takes place, especially in the colon, in hysteria." Occasionally when the paroxysms are intense, and there is no symptom of general or local excitement, he gives from thirty to fifty drops of laudanum. Dr. Roots adopts the old opinion that one of the beneficial results of nauseous medicines, such as valerian, is the peculiar effects they produce on the mind, similarly to the effect of feathers burned under the nose. Tonics, as iron or quinine, should not be used unless called for by the absolute debility of the constitution.

The next case is one of *compound dislocation of the sternal end of the clavicle inwards*, produced by the sharp end of a pickaxe which was driven in by a fall of earth. When the finger was introduced into the wound, the great pectoral muscle was found to be torn entirely from its clavicular attachment, and the finger could be passed as far outwards as the coracoid process of the scapula, and inwards it followed the clavicle to the trachea, on the right and forepart of which it rested, sunk behind the upper bone of the sternum, so that it slightly affected respiration and deglutition. The shoulders were brought back with straps attached to a back-board, and the bone readily resumed its place; the elbow was brought forward and bound to the side. The patient, who was under Mr. Tyrrell's care, got well without any untoward symptom.

The next is a case of *Chronic Dysentery*, reported by Mr. W. Wegg, and illustrated by the clinical remarks of Dr. Roots. The patient was a sailor, and he had contracted the complaint in the South Seas. There was purging of mucous stools mixed with blood, pain in the abdomen increased by food and pressure; thirst; tongue coated; red at the tip and edges; pulse 108, weak; emaciation. The case proved very obstinate. In the first twenty-six days leeches were applied, opium and starch injections given; the patient was put on a farinaceous diet, and the mouth was touched with mercury, combined with opium; but, although the secretions were improved, the disease was not cured.

The compound powder of kino with sulphate of copper, and afterwards with sulphate of iron, were fairly tried, but the mineral astringents increased the irritability. Subsequently ten grains of nux vomica were given every six hours, for three days, but it was discontinued from its producing nervous symptoms. This treatment occupied four months. Dr. Roots then resolved to use vegetable astringents only, and prescribed Infus. Gallæ, ℥iss.; Pulv. kino comp. gr. xxv., sextis horis. The symptoms improved, and in two months he went out, looking fat and healthy; the dose of the medicine having been gradually diminished.

Dr. Roots, in his remarks, states that he has not found mercury, even in conjunction with opium, counter-irritation, and local depletion, cure chronic dysentery; but, on the contrary, the irritability of the intestines has often been increased by it,—an observation which coincides with our own experience. He, of course, never uses astringents when there is much febrile action; but, after lessening inflammation and spasm by the ordinary means, he resorts to them; and when the disease becomes more chronic it is the best practice, even when there may be sufficient tenderness and pain to require leeches from time to time, and counter-irritation. Although it failed in this case, Dr. R. has often found half grain doses of sulphate of copper (combined with opium and other astringents) very useful in relieving the chronic inflammatory action of the mucous membrane. Sulphate of iron also is a useful addition, possessing tonic as well as astringent powers; whereas the copper is purely astringent. In some rare cases, Dr. R. has found nux vomica succeed where the usual astringents have failed. The infusion of galls he occasionally uses when other remedies have been unsuccessful, and with considerable advantage. It is, however, so nauseous, that few patients will submit to it. We wonder Dr. Roots has not given a fuller trial to ipecacuan in this disease. He cannot be unacquainted with the ancient fame of this remedy; and it has been recently most strongly recommended in this disease, by Mr. Twining, in India. In chronic dysentery, Mr. Twining employed the infusion of ipecacuanha.

The next is a very valuable paper on *Diseases of the Joints.* A series of cases are reported by Mr. White and commented on by Mr. Tyrrell. First of all, four cases are reported of inflammation of the fibrous capsule of the hip-joint, and Mr. Tyrrell enters at length on the rationale of the symptoms, showing how they point out the seat of the disease, its form, and the texture affected. The chief symptoms are pain, flattening of the buttock, and apparent lengthening of the limb. In three of the cases there was pain in the anterior part of the hip-joint and anterior and inner part of the knee.

"The pain at the anterior part of the joint denotes here that the inflammation or morbid affection is confined principally to the corresponding part of the capsule, and this is again indicated from the sympathetic pain affecting the knee: it is upon the anterior and inner part of the knee that the patient feels pain." (P. 285.)

The flattening of the buttock is thus explained:

"As soon as the capsular ligament becomes affected, we have sympathetic pain induced from nervous communication, and also sympathetic affection of the muscles; so that those employed in effecting the movements of the leg or thigh in a degree lose their power, and become incapable of performing their duty to the proper extent. It is solely to the loss of power in the muscles, that this flattening of the glutei (which principally form the mass of muscle in the buttock) is to be attributed." (P. 286.)

The question of the lengthening or apparent lengthening of the limb, a circumstance which has been the subject of so much investigation and difference of opinion among surgeons, is discussed at some length by Mr. Tyrrell. His views being somewhat novel, we give them at length, and recommend the reader to compare them with those maintained by Mr. Wickham, in his very excellent work on Diseases of the Joints, p. 146.*

"A person who is the subject of diseased hip is always relieving the affected side, and he, therefore, gets a habit of throwing the pelvis in an oblique direction. Usually, the affected leg is advanced forwards and made to rest lightly on the ground, the weight of the body being supported on the sound limb. There is a difference in the height of the cristæ of the ilia: the sound side is thrown an inch or more above the affected side. This, after a time, remains when the patient is recumbent; so that, when you put the legs together to make an accurate examination, you find the same apparent difference in the limbs as when the patient is erect. But there is another circumstance, and a very important one, which gives rise to an apparent lengthening of the limb, when the patient is recumbent. The capsular ligament which comes from the acetabulum embraces the neck of the thigh bone; but as this joint admits of motion in every direction—abduction, adduction, flexion, extension, rotation, and the intermediate movements, we know that the capsule is longer than is absolutely necessary to retain the articular surface of one bone in contact with another; so, if you strip off the whole of the muscles from the capsular ligament, you may then draw the thigh bone from the acetabulum to the extent of an inch or more. The capsular ligament does not proceed in a straight line from one point to another; and when the limb is in a straight position, the capsule forms a sort of loose bag, not projecting out, but it is longer than the mere exact distance, or a straight line between the points of origin and insertion.

"The head of the bone is kept against the acetabulum in a healthy state, by the influence of the surrounding muscles. All the muscles which pass over the hip-joint have more or less influence of this kind; but more especially those close on the articulation. As we find the glutei lose their power, so the other muscles about the joint also lose their power; and if by force (when the patient is recumbent) you draw together the two legs, the separation of the head of the femur from the acetabulum

* A Practical Treatise on Diseases of the Joints. By W. J. Wickham, Surgeon to the County Hospital, Winchester.—London, 1833.

takes place on the injured side, from the muscle not affording resistance, and a difference is made in the length of the two legs in consequence of the separation of the articular surfaces on one side, to the extent of an inch or more." (P. 286.)

The wasting of the limb partly proceeds from the same cause; the muscles lose their tone principally by sympathetic influence, and become flaccid, and therefore the diameter of the affected limb is less. After a time the muscles, from want of use, diminish. Mr. Tyrrell thinks the *kind* of pain experienced in these cases points to the seat of the disease.

"The character of the pain itself, being aggravated at night, is indicative of the disease attacking fibrous tissues. We see this very clearly in the inflammation of the sclerotic coat of the eye." . . . . "It is an intermittent or remittent form of pain, of a peculiar kind, being dull or aching, not lancinating nor sharp. The pain is not so severe but that the patient may bear it, but it is sufficient to disturb his rest, and therefore it produces exhaustion." (P. 289.)

In regard to the treatment of diseases of the hip-joint, Mr. Tyrrell, with us, differs from the practice recently recommended by Mr. Coulson. Although in the earlier stage he approves of milder treatment, trusting a good deal to rest; yet when the case is at all severe and well marked, he advocates, from experience, the use of active local measures, particularly recommending them to be applied as soon as possible to the affected part.

"Where the symptoms are slight, and the case is incipient, I have often found that the application of a blister on the surface, combined with attention to the secretions, and rest, produced all the good that I could wish. If the disease has been of long duration, and you think, from examination, that it is of some extent in the fibrous tissue, it is better to resort at once to a more severe form of counter-irritation, such as the application of a moxa, or nitric acid, or potassa fusa, or any other mode of establishing an external wound. The moxa is a good mode of exciting counter-irritation; and I know that, where patients have had it created by various means, by moxæ, by nitric acid, and potassa fusa, they have given a preference to the moxæ over every other plan. It is easy of application; but if patients object to it, you may use nitric acid or potassa fusa, or any of these, for they will have the same beneficial effect. And it is not my practice here, and it is the result of experience, to keep the moxa open by the use of an extraneous matter, so as to form what is usually denominated an *issue;* nor is it my practice to keep open a blister." (P. 291.)

We are sorry that we cannot enter upon the subject of diseases of the articulating extremities of bones, and of the ligaments, particularly the lateral ligaments of the knee-joint. Both these subjects are illustrated by cases which are most ably commented on by Mr. Tyrrell.

Dr. Williams advocates the tonic treatment of erysipelas. Three cases treated by him with wine are reported by Mr. Bullock; two of erysipelas of the head and face, and one of the leg and thigh. The following is Dr. Williams's mode of treatment:—

"The patient is put on a milk diet; the bowels gently opened, and from four to six ounces of port wine, together with sago, allowed daily. This mode of treatment it is seldom necessary to vary throughout the whole course of the disease, and the delirium, if present, is generally tranquillized, and, if absent, prevented. The tongue rarely becomes brown, or only continues so for a few hours, and the local disease seldom passes into suppuration or gangrene. In a word, all the symptoms are mitigated, and the course of the disease shortened. I have pursued this treatment for several years, and I hardly remember a case in which it has not been successful." (P. 336.)

Dr. Williams takes some pains to show that this treatment with wine is justified by theory as well as by practice; but we do not think he has succeeded. He begins by stating that inflammations which depend on morbid poisons require a treatment different from those arising from mechanical or chemical causes; in the former cases, the constitution has received a shock on depression which, as a general principle, is aggravated by bleeding, so that the system is more exposed to the influence of the poison. He then brings forward the strong evidence of Dr. Wells, Pitcairn, and others, to show that erysipelas is a contagious disease, depending on the agency of a morbid poison. If this is admitted, says Dr. Williams, it follows that bleeding, as a rule, is inadmissible. The way in which this axiom is proved, is one of the most inconclusive specimens of medical reasoning we ever recollect to have met with; and, in justice to Dr. W., we give it in his own words.

"An experiment suggested by Magendie was repeated here a few months ago, in which two rabbits, the one having been bled to as large an extent as his small blood-vessels would admit, and the other left untouched, were poisoned by strychnia. The one that had been bled died in twenty minutes, while the other survived for forty-five minutes. This experiment has been repeated by so many different physiologists, and so uniformly with a similar result, that it seems to be a well-established law that bleeding, in all cases of poisoning, the more certainly lays the system under the action of the poison, and aggravates all its consequences. All analogy, therefore, would lead us to conclude that, in the treatment of erysipelas, bleeding, if in any case admissible, must be the exception and not the rule." (P. 328.)

The practical conclusions which this physiological experiment appear to us to justify are, that if a patient in a healthy state was largely bled, and consequently much debilitated, he would be more likely to become attacked by erysipelas if exposed to its contagion. But it no more proves that, when inflammation already exists in the debilitated body, the antiphlogistic treatment is not required, than it proves the impropriety of treating with antiphlogistics the inflammation of the stomach which frequently follows poisoning by arsenic or other irritants. Analogy with other diseases, instead of supporting Dr. Williams, refutes him. He places typhus fever in the same category with erysipelas; but, if inflammation of the lungs, for instance, occurs in the course of typhus fever, a modified antiphlogistic practice is the only one which gives the patient any chance of recovery. This indiscriminate recommendation of one kind of treatment in every case for a disease whose character varies so much under different circumstances, is injudicious. If Dr. Williams's pupils leave him with the impression, that the simple treatment of from four to six ounces of port wine daily, given to that class of patients who are suffering from erysipelas in the wards of a London hospital, (and the old wards of St. Thomas's, according to Dr. W., are crowded and close,) is to be invariably successful in their country practice, they will have imbibed both incorrect and injurious notions. The want of unanimity among medical men as to the best treatment of erysipelas, whilst it is an argument that the disease is not thoroughly understood, may also be accounted for by the changeable and varying character of the disease; and whosoever prescribes for the mere name of this complaint, will surely go astray.

The concluding paper is by Mr. Travers on the subject of *Tumours*, and is written with his usual ability. With the following remarks on errors in diagnosis we entirely coincide; and we have no hesitation in saying that a logical and faithful book on this subject would be of more real value to the practitioner in surgery than the hundreds of volumes which every year presents to us, the offspring of a blind empiricism, and as short-lived as the reputation of the never-failing remedies which they so boldly and so lavishly promulgate.

"There are numberless examples of faulty diagnosis in the practice of the most reputed surgeons, and this has been as often proved by the decision of the patient, as by that of a consultation. Influenced by hope or fear, the former has clung to the chance of life held out by an unadvised operation, from which no relief was expected, or resisted its performance, although urged on all hands to embrace it, as the last resource in his extremity, and has survived and recovered. So a charlatan has not seldom carried off the credit of a cure, speculating boldly on the doubts of the honest practitioner, and the credulous confidence of the sinking patient. A register of 'errors of diagnosis,' faithfully recorded, would be a source of curious, and not unprofitable, information. But the discriminative faculty being the touchstone of scientific and practical skill, upon which every individual, aware of its value by implication and in effect, especially plumes himself, the pride natural to man interposes an obstacle, which only a single and intense preference of truth to all other considerations can effectually overcome." (P. 344.)

The first article in the fourth Number of St. Thomas's Reports is a useful clinical lecture, by Dr. ROOTS, on *Phlebitis.* This is followed by a case of *Stone in the Bladder*, with remarks, by Mr. TYRRELL, who details minutely every step of the operation. This is indeed the only plan by which the hospital student can gain much instruction, as the most important part of lithotomy is performed out of sight. He makes use of the beaked knife, and tells us that he chose this instrument after performing the operation on the dead body repeatedly with all the other instruments, and afterwards dissecting the parts. He objects to the gorget, because it requires great force, and because the incision is insufficient for a large stone; he found that with the common scalpel (employed by Mr. Key,) he could make an incision rapidly and sufficiently extensive, but that he wounded the fundus of the bladder, when that organ was contracted, which cannot be done with the beaked knife. The success which he has had is the chief recommendation ; for, of forty-one cases of lithotomy which he has performed in public and in private since he has been in the profession, only one has terminated fatally; and this was the case of a little boy, about three years of age, from whom he removed a small stone without difficulty, but who died from erysipelas, at the time prevalent in the ward. The patient, whose case is the foundation for Mr. Tyrrell's remarks, also had erysipelas and peritonitis, from both of which he escaped. The treatment of these consecutive diseases is ably discussed. The majority of cases of erysipelas in St. Thomas's Hospital are of patients who have suffered from injury, and who require the tonic treatment. Ammonia is the remedy on which Mr. Tyrrell relies, but he does not partake of the exclusive views of his colleague to which we have alluded; for, if the pulse is full, bounding, hard, and incompressible, with a foul tongue, he bleeds, purges freely, and keeps the patient on an abstemious diet. In traumatic peritonitis, frequently an effect of lithotomy or the operation for hernia, he places his chief reliance on calomel and opium. From the great influence of mercury in checking inflammatory affections of the eye, which quickly go on to a destructive termination in consequence of adhesive deposit, he, in common with other practitioners, was naturally led to carry out the principle to other textures liable to the same consequences, as the serous membranes. In weak and feeble habits, Mr. Tyrrell resorts to this alone without bleeding, and he has saved many whose powers would have been prostrated by the abstraction of blood. He gives two or three grains of calomel and one-third of a grain of opium every three or four hours, until the mouth is affected. The whole of this lecture will amply repay an attentive perusal.

Dr. Roots makes some remarks on *Pericarditis*, but they throw no additional light on that obscure disease. He omits to mention as a symptom the altered sound on percussion, one of the most valuable. He recommends bleeding and mercury; and has had of late years greater success than formerly, by keeping his patients for a greater length of time under the influence of mercury, and employing local bleeding if there was any increased action of the heart or bellows-sound, when the active symptoms were subdued: for he found that his patients were very subject, on exposure or slight irregularity, to relapses, which he attributed to some degree of inflammatory action going on insidiously in the lining membrane of the heart.

*Chronic Laryngitis* is the next subject which Dr. Roots notices, and his observations on the treatment of the disease are, as usual, highly judicious. Where there is much irritability of the mucous membrane of the larynx, he avoids ipecacuanha; for, from the excessive excitement which it sometimes produces when an atmosphere impregnated with it is accidentally inhaled in perfect health, he considers it a stimulant, and rather applicable to chronic coughs attended with great debility and great relaxation of the bronchial membrane. He gives a case in which the inhalation of ipecacuanha produced irritation of the bronchial membrane, requiring active bleeding; and our own experience affords more than one instance of the intense irritation produced by the accidental inhalation of this drug.

Three cases of *Medullary Sarcoma* are next given, followed by some remarks, by Mr. Travers, on the analogies between scrofulous and medullary depositions; a subject which he has ably discussed in the Medico-Chirurgical Transactions.

The last article is one by Dr. Roots, on *Enuresis*. A girl, æt. 16, had no pain, or uneasiness, or urgent necessity to pass her water, but it came away involuntarily at night, and whenever she made the least exertion: she only retained it when sitting perfectly still. Dr. Roots considered it to be owing to a torpid state of the sphincter of the bladder: he applied a blister to the sacrum, and ordered fifteen minims of tincture of cantharides every six hours. She was relieved in a few days, and discharged in three weeks. The case was of five years' standing.

This Number completes the first volume of these Reports, and is furnished with a table of hospital formulæ which have been referred to. We trust that all the medical officers will continue to make it the vehicle for authentic reports of their clinical lectures; by so doing, they will enable their former pupils to benefit by their increased experience.

Mr. Key commences the third Number of the Guy's Hospital Reports with "*some Observations on the Nature and Treatment of Ganglion, Bunion, &c.*" He has found, on dissection, that ganglia, such as are formed about the carpus on the flexor and extensor tendons, consist of a double bag, the outer one tendinous and firm, the inner, like a synovial membrane, thin and secreting. Their contents are not generally like synovia, but like the outer layers of the crystalline lens. Mr. Key prefers puncturing them with the point of a lancet, (or cataract needle, when the tumour is small,) to the common plan of dispersing them with a smart blow. It is more sure, less painful, and (in his experience) the cyst is little disposed to inflame; the closure of the opening by sticking-plaster preventing it. Puncture is the only remedy for the small tumours at the base of the palmar side of the fingers. Blisters are slow and often fail, and blows cannot be accurately aimed. Pressure should be kept up for some time afterwards. The ganglion patellæ, or housemaid's knee, Mr. Key treats in the same way; and, if this is not permanently beneficial, he finds the seton, of a few threads, the most ready, mild, and effective remedy; less annoying even than a blister. The seton is more especially adapted to those ganglions which have become indurated and almost solid from successive depositions of adhesive matter on the cyst, from continued inflammation, which sometimes contain loose bodies like melon-seeds.

"Fortunately, the seton is equally successful in promoting suppuration of the cyst as it is in the smaller and soft ganglia. But the most gratifying result is the entire disappearance of the hardened coats of the cyst by absorption. The indurated parietes appear to be produced, and to be kept up, by the irritation of the bag; which being filled up by the inflammation and suppuration established by the seton, ceases to act as a cause of irritation, and the absorbents set to work for the removal of the walls of the tumour." (P. 420.)

Ganglia of the foot, of which the bunion is the most frequently met with, generally depend on undue pressure on some parts of the tarsus; and Mr. Key prefaces his remarks on bunions by some useful observations on the effects of pressure on the feet. The drawing, No. 3, exhibits one species of deformity, which was witnessed in a young gentleman who had been in the habit of wearing boots so short as to compress the foot longitudinally.

"The foot had been thus made to assume a curved direction: the arch of the tarsus had been considerably increased, producing a large projection on its dorsum, and a corresponding hollow in the sole, rendering the foot altogether shorter than nature had intended it to be. The altered form, however, was the least part of the inconvenience; for the act of progressing had become uneasy and cramped. By the unnatural strain created upon the top of the arch at the junction of the os naviculare and os cuneiforme internum, the ligaments had been inflamed, and had become thickened; not, as in the case of the patella, forming a bursal cavity, but giving rise to a painful and indurated swelling, which gave pain under the pressure of the boot, and rendered the foot stiff and uneasy in the act of progression." (P. 423.)

A more aggravated instance of the same kind of deformity was produced in a country boy, who was compelled to wear shoes cased with iron round the edge of the sole. The shoes did not wear out, but the feet continued to grow. The tarsus was thus more arched than usual; and the phalanges formed angles with the metatarsal bones, being thrown upwards so as to make the balls of the toes more than usually prominent. The great toe was very much distorted upward; its ball was thus exposed to great friction, and in consequence an intractable ulcer formed; for which he was admitted into the hospital.

Mr. Key believes that bunion is the effect not so much of pressure from the shoe, as of excessive weight received on a weak tarsus and metatarsus, occurring in young persons who take exercise on foot disproportioned to their strength. The tarsus in these cases first becomes flattened, in consequence of the ligaments, and probably the bones also, yielding to the superincumbent weight.

"If the pressure be continued, the under part of the arch at length nearly comes in contact with the ground, and the patient becomes flat-footed. On the outer part of the tarsus, a corresponding change is perceptible: its upper surface becomes depressed and hollow, and thus a general appearance of deformity and awkwardness of gait are the result. I have seen the young among the female part of many families gradually acquiring this tarsal deformity, from the excess of exercise which they were enjoined by their parents to take; and, in truth, while the error of our forefathers led them to confine girls within too strict limits, and to allow them too little use of their limbs, that of the present day runs into the opposite extreme; and many a young person has the proportions of her feet spoiled, and the foundation of bunion laid, by being kept on her feet for hours, without rest or intermission. In some, the deformity is confined to a slight projection of the head of the astragalus, which has weighed down the inner strong plantar ligament. This gives an uncouthness to the walk of the girl, and renders the foot unsightly, but produces no other effect. The change of shape is not long confined to the tarsus; the joints of the toes suffer next, especially that of the great toe. The ligaments of this joint being strained in common with those of the tarsus, at length yield, and the toe is gradually forced outwards by the oblique bearing of the foot on its inner plantar surface. It is rarely that we find persons whose tarsal arch is flattened with a great toe in a line with the foot: it is usually inclined outwards, so as to form an angle with the metatarsal bone. The inner part of the joint thus forms an angular projection; slight at first, but becoming more and more prominent as the pressure increases, and the joint continues to yield. . . . In persons among the working classes, the distortion is sometimes so great that the great toes form nearly a right angle with the metatarsus." (P. 425.)

This kind of deformity gives rise to bunion, and it cannot be counteracted too early. The best contrivance Mr. Key has adopted is the following:

"The offending toe is placed in a separate compartment of the stocking, like the finger of a glove: this again is inclosed in a separate part of the shoe, which is contrived by fixing a piece of firm cow-leather in the sole of the shoe, so as to form a separate apartment for the toe. By these means it is kept in a straight line with the foot, or parallel to its fellows; and, the pressure against the inner side of the joint being removed, the joint acquires a sufficient degree of strength to enable it, in a few months, to dispense with the artificial support." (P. 427.)

Patients who have suffered previously extreme pain have walked well with such a shoe. Where matter forms, the abscess should not be opened with a knife. Mr. Key has known gangrene of the foot and death ensue from opening an inflamed and suppurating bunion; and in three cases exfoliation of the bones, with a most tedious and painful suppuration of the surrounding structures, took place.

It is pleasing to see hospital surgeons thus giving the result of their experience in the less showy branches of surgery, (descending even to corns;) for the young surgeon is often more at a loss in such little matters than he would be in undertaking those rarer cases of disease, to which, from their scarcity and magnitude, he has paid too exclusive attention.

The deformities and little complaints of the feet are of great importance, from the multitude of the sufferers. In all, or almost all adults, the beauty which depends on perfect form is impaired. The foot of an infant yet unfettered looks almost as if it belonged to a different species to that of a full-grown person; but the loss of beauty would be a matter of less moment, did not the deformity lead to much inconvenience and pain. All classes of shoe-wearing people are subject to these infirmities: the richer people are cramped for the sake of appearance, the poorer from ill-made heavy coverings. On the contrary, the Irish poor, whose poverty relieves them from such incumbrances, have almost universally well-formed feet, and their perfect adaptation to every end is as striking as the more frequently quoted instance of the fitness of the camel's hoof for his desert journeys. Among recruits, the Irishman's foot is remarkable for its superiority of form. Mr. Key has very ably pointed out the way in which bunions are produced, not so much by pressure as by over-exertion, and there can be no doubt that this deformity may be caused by undue exercise in weakly girls, who are suddenly put upon a hardening system disproportionate to their strength and former habits; but we think that he has over-rated this cause. Our own experience has not made us fear that any great number of young people are in the present day likely to be impaired in their proportions by too much exercise enforced as a task. Perhaps sufficient attention is not paid at an early age to the manner of walking; for a careless slovenly way of using the feet is particularly injurious to their form. The beauty of the feet of the ladies of Madrid is proverbial: they make walking their study, and impart to it an unrivalled grace. Even the lower orders of the Parisian females pay much more attention to the decoration of their feet than is to be seen in a similar rank of life in this country: they are consequently more careful in their walk, and the form of their feet is much superior to that of the lower orders here. We were much struck with this fact in the Paris dissection-rooms.

The next paper is by Mr. T. W. King, "*on the Thyroid Gland, with Observations on the same Subject, by* Sir Astley Cooper."

It appears, from notes taken by a pupil of Sir A. Cooper's lectures in 1798, and from notes which he made of experiments in 1826 and 27, that he then described the structure of the thyroid gland as laminated, and consisting of cells (not of granules) containing a fluid. He remarked that he had found absorbents as large as a crow-quill going to the thoracic duct, and he thought it probable, as the gland has no duct, that the absorbents perform that office, and convey the fluid into the thoracic duct. Mr. T. W. King has very recently dissected the thyroid gland, and, without being aware of the previous discoveries of Sir Astley, has confirmed his opinions, distinctly proving the laminated arrangement of the lobules, the cellular structure of the gland, the existence of a peculiar fluid, and the transmission of this secretion through the absorbents to the great veins of the neck. The fluid appears like weak gum, and coagulates without opacity in alcohol and by heat. Its chemical composition has not been satisfactorily ascertained, but the effects of various reagents upon it are minutely detailed. Sir A. Cooper, with his usual candour and kindness, gives Mr. King due credit for accurate and minute observation, and signifies his intention (as the subject is still open to much investigation,) to pursue the enquiry in conjunction with him. In a literary point of view, Mr. King's paper is so defective, its arrangement so faulty, and the style so obscure and inflated, that the co-operation of a more practised writer and physiologist will add greatly to the usefulness of any further investigations. Some beautiful engravings on zinc by Mr. Canton display the laminated form, cellular structure, &c. of the gland.

Sir Astley Cooper next relates some *Experiments on tying the Carotid and Vertebral Arteries, and the Pneumo-gastric, Phrenic, and Sympathetic Nerves.* It is important for the practical surgeon to ascertain the effects produced by the obliteration of large arteries on the functions of the parts which they supply, as well as the mode by which nature remedies the defect; and in no department has modern surgery more obviously advanced than in this, the boldest operations having been planned, and successfully performed, under the guidance of principles founded on well-ascertained pathological facts and direct experiments. It is unnecessary to mention that Sir Astley Cooper has contributed largely, both practically and experimentally, to the more accurate information we possess on this subject, and he is still found anxious to throw more light on those points which are yet obscure. Our limits compel us to omit the details of the experiments, and to confine ourselves to the inferences.

It is well known that one carotid has been frequently tied in man, and even both, at distinct periods, without affecting the functions of the brain. Dr. Baillie found the carotids obliterated by disease. Dogs and rabbits are but slightly disturbed by the ligature of both the carotids, and speedily recover perfectly. But it is not so with the vertebral arteries, which seem to be more directly connected with the supply of blood to the brain. The effect of tying these arteries in rabbits is to render their breathing immediately laborious. The animal becomes dull, indisposed to exertion or to take food, and never survives more than a fortnight. On account of their importance, their course is securely defended by bone, otherwise pressure might cause sudden death; they are tortuous, to prevent too sudden a rush of blood to the head; and, as they pass through foramina of bone, any great increase in size is prevented. Compression of the carotid and vertebral arteries of the rabbit at the same time, by applying the thumbs to both sides of the neck, the trachea remaining free, produces cessation of respiration. This fact is more strikingly seen by applying ligatures round the four vessels, and tying them simultaneously, when stoppage of respiration and death immediately occur. If this is done in the dog, it loses its volition and sensation, and appears as if intoxicated; but the anastomosing vessels gradually restore the circulation, by means of the other branches of the subclavian artery at the back and sides of the neck. As, in these experiments, the pressure on the nerves might influence the result, Sir Astley wished to know the effect of ligatures upon them only. He first tied the pneumo-gastric nerves: the animal lived *twelve* hours; its lungs were loaded with blood, and venous blood was found in the carotids, and circulated in the arteries some time before death, the blood gradually becoming less arterial; yet the heart continued to beat. There was a remarkable diminution of animal heat. Was this owing to a cessation of the chemical process by which arterial is changed to venous blood? or to a want of supply of arterial blood to the nerves? or to both causes? When the animal had eaten after the operation, the œsophagus contained food, owing to its being paralyzed; the stomach was full, from the arrest of the digestive function. "This nerve, then, is most important: 1st, in assisting in the support of the function of the lungs, by contributing to the changing of the venous into arterial blood; 2dly, in being necessary to the act of swallowing; 3dly, in being very essential to the digestive process." On tying the phrenics, the most determined asthma was produced; breathing proceeded by the intercostal muscles; the chest was elevated to the utmost by them, and in expiration it was as remarkably drawn in. The animals did not live an hour, but did not die suddenly, as when the carotids and vertebrals were tied. The blood in the carotids became venous, but the lungs were not congested. Little effect was produced by tying the grand sympathetic: one rabbit was kept seven days, and then killed, and one nerve had ulcerated through. The other animal, on which the operation was performed above a month previously, was still living at the date of the paper. Lastly, Sir A. Cooper tied the phrenic, pneumo-gastric, and sympathetic in each side; and the animal lived about a quarter of an hour, and died of dyspnœa. "The sudden death, then, that takes place from pressure at the sides of the neck must not be attributed to an injury to the nerves, but it is owing to the impediment to the due supply of blood to the grand centre of nervous influence."

DR. ADDISON'S "*Observations on fatty Degeneration of the Liver*" are of considerable interest, as they are the first attempt which has been made towards ascertaining this disease during life. Fatty degeneration of the liver has, on this very account, attracted but little attention in this country. M. Louis first pointed out its connexion with tubercular disease of the lungs; for, in 120 cases of phthisis, he met with forty-four instances of this complication. In every case of fatty liver he has seen, there was more or less tubercular disease of the lungs. Dr. Addison's own experience is against this connexion being uniformly the case, at least in this country: within the last two months he has met with three instances of fatty liver without any vestige of tubercle either in the lungs or in other parts of the body; and in two of them no suspicion had been entertained of disease either in the lungs or liver, both individuals having remained plump and fat to the time of their death: one died of organic disease of the brain, and the other of pericarditis succeeding an attempt at suicide by cutting the throat.

The appearances of the fatty liver are thus accurately described, and a coloured engraving on zinc increases the clearness of the description.

"It is observed to be of a cream or pale-yellow colour, figured irregularly with brownish or deep-orange spots. It is usually, though not always, more or less enlarged, and sometimes very considerably so. When cut into, its interior is found to present an appearance somewhat corresponding to that of the exterior: excepting that the brown and pale-yellow tissues are much more uniformly distributed throughout the entire substance of the organ than they are upon its surface. It is sometimes softer, and more readily crushed between the fingers than is the healthy liver: sometimes, however, it is firmer than natural, and occasionally even of a scirrhous or almost horny hardness. It will often very perceptibly grease the scalpel, or impart an unctuous feel to the fingers; and, on being exposed to the flame of a candle, will yield a considerable quantity of fat." (P. 477.)

An analysis was made of a portion of fatty liver by Mr. G. Bird, and each pound of liver was found to contain 333.2 grains of a soft brownish fat.

The value of the following laudable attempt to connect this disease with an external sign must depend on its confirmation in more numerous instances, and by other observers.

"Having, in the course of my experience, been often struck with a remarkable appearance of the face in certain patients—an appearance dependent not so much on the expression of countenance, as the texture and aspect of the integuments,—and having observed the exact resemblance of the appearance in each case, I endeavoured to connect it with some corresponding uniformity in the accompanying disease; and at length arrived at the conclusion that, when strongly marked, it is indicative, if not pathognomonic, of fatty degeneration of the liver. It is purely integumental; as it is not confined to the face, but may pervade the whole surface of the body; although I am disposed to think that it is earliest observable, as well as most conspicuous, in the integuments of the face and backs of the hands. To the eye, the skin presents a bloodless, almost semi-transparent and waxy appearance: when this is associated with mere pallor, it is not very unlike fine polished ivory; but when combined with a more sallow tinge, as is now and then the case, it more resembles a common wax model. To the touch, the general integuments, for the most part, feel smooth, loose, and often flabby; whilst, in some well marked cases, all its natural asperities would appear to be obliterated, and it becomes so exquisitely smooth and soft as to convey a sensation resembling that experienced on handling a piece of the softest satin. Whether this condition of the integuments precede or follow that of the liver, and whether the two are necessarily associated in every instance, I am by no means prepared to offer a decided opinion; but having, on several occasions, confidently and correctly predicted finding the fatty liver, from having observed the particular condition of the integuments just described, I have ventured to make the crude fact known to the profession, in the hope, that, attention being once directed to such a starting point, something more interesting, and of greater practical utility, may result from its further investigation." (P. 479.)

The matter and manner of Dr. Addison's short communication make us regret that he has not more largely contributed to this number of the Reports.

We must confine ourselves to the numeration of the titles of the next four papers, as they either consist of isolated cases, without remarks which can be separated from the cases themselves, or they are incapable of abridgment. This latter observation applies to a clever paper of Mr. Golding Bird, on "*Cystine or Cystic Oxide*," and to "*the Case of a large bony Tumour in the Face completely removed by spontaneous Separation; to which are added, Observations upon some of the Functions of the soft Palate and Pharynx;*" by Mr. Hilton. The other reason applies to "*Cases occurring in the Clinical Wards;*" and to "*Cases of Hernia, Popliteal Aneurism, wounded Ulnar Artery, and Malignant Tumour, with Remarks,*" by Mr. Bransby Cooper.

A long paper (of fifty pages) on *Chlorosis and its Complications*, by Dr. Ashwell, comes next. Its perusal leaves the impression either that Dr. Ashwell does not think clearly on the subject he writes upon, or that he is unable to express his notions with simplicity and precision, or both. Indeed the verbosity and diffuseness, together with the want of lucid order, seem to show that the imperfection must depend on both of these causes. This is the more to be regretted, as there is internal evidence in the paper that Dr. Ashwell has seen much of the disease in question, and (with regard to treatment) is a sensible and judicious practitioner. He is of opinion with some French physicians—and we think justly—that chlorosis depends on a morbid condition of the blood, and that it does not necessarily spring from amenorrhœa; in the treatment, he advises the cautious use of purgatives and iron. This part of his paper is the best; but he afterwards confuses the subject much by discussing under the head of "complications of Chlorosis" what are usually considered as forms of hysteria. Thus, the anomalous headaches of young women, commonly, after the example of Sydenham, regarded as forms of hysteria, are called "complications of chlorosis from a functional affection of the cerebrum;" and this is an example of the lax way in which Dr. Ashwell employs words. It is not known whether such pains depend on the cerebrum, or cerebellum, or membranes, or bones or nerves supplying the parts above them; probably they sometimes depend on affections of each of these parts; and yet the word "cerebrum" is used, fixing the functional affection to one part out of very many, without any good grounds. The paper also is full of "prettinesses," which become by their frequency offensive. The blood is almost always "the vital fluid;" diseases susceptible of cure "come within the scope of medical agency;" "the change from girlishness to womanhood is to be accomplished; puberty is viewed as the artificer of this transition." Of a weak girl becoming stronger at this period it is said "perchance, some of the former delicacy may be merged in the slight increase of vital energy which has been evinced." When the girl becomes healthy "the *tout ensemble* of the disease is gone;" if she becomes highly nervous, this is "the bequest of a very protracted chlorosis." If every page had not abounded with such sentences (some of them put in italics,) we should not have alluded to them; for we always find much more pleasure in dwelling on what is really valuable. To a beginner unacquainted with disease we would not recommend this essay: yet we willingly allow that there are some hints, particularly as to the danger of functional passing into organic disease, well worth attending to: some of the cases are also very instructive.

We pass on to a paper of Dr. Bright, on *Jaundice*, which is an admirable specimen of what such essays should be. It is full of valuable matter, clearly conceived and arranged and well expressed. No one can read it without feeling that he has a more methodical and practical knowledge of the disease than he had previously: if the facts which he had collected by reading and actual observation had not been before classified in his mind, and, from his inability to arrange them, he had found them all but useless, he will now admit that they fall into their proper order, and he can more clearly understand cases which were previously involved in much obscurity. It is not intended to be implied that Dr. Bright's paper will completely elucidate the numerous and complicated causes of jaundice, which is only a symptom common to numerous diseases, and not a disease itself; but, by a simple arrangement of the diseases producing it, the information we possess is rendered more available. The paper is so complete in itself, and there are so few details which can be omitted without injuring the sense, that it is impossible to do it justice in our confined limits. We must content ourselves with a mere specimen of it, earnestly recommending its entire perusal to the reader. If the writer of this excellent monograph proceeds in the career which he has hitherto so successfully pursued, he will secure to his name a place among the most honoured of British physicians. We were gratified lately by observing, in a Swedish journal, the disease of the kidney, first described by him, denominated *Morbus Brightii*. We think it probable that the plurality of Dr. Bright's pathological discoveries will soon render this term logically incorrect.

"The causes which generally give rise to Jaundice will, perhaps, admit of the following classification:—1. Congestion of blood in the liver. 2. Obstruction of bile in the biliary ducts, and more particularly in the larger ducts. 3. Chronic change in the structure of the liver. 4. Inflammatory action of the liver." (P. 604.)

The chief object of this communication is to illustrate the fourth class, and to this part of the paper we must confine our extracts.

Dr. Bright regards "a state of inflammatory action more or less generally pervading the liver," as one of the most common causes of jaundice. He considers this as differing essentially from the chronic action described under the third head, inasmuch as in the former the secretory portion rather than the connecting cellular tissue is affected; while the causes are also different, being, in the fourth variety, irregular diet, exposure, external violence, the irritating effects of biliary concretions, and of retained bile, and perhaps of mercury. Its progress varies.

"It frequently comes on very insidiously, with symptoms and feelings of general constitutional derangement, depression of spirits, slow pulse, oppressed breathing, wandering abdominal pains, constipated bowels, and sometimes sickness of the stomach. In a day or two, the conjunctiva becomes tinged; and in a few days more, there is universal bright bilious suffusion of the skin." (P. 614.)

"In other cases, the inflammatory action is attended with much more severe symptoms, with considerable pyrexia, quick pulse, flushed countenance, and dry tongue, while a jaundice of the most intense colour is diffused over the whole surface. The stools are, both in the more and less acute cases, of a light colour; but less decidedly so, and subject to greater variations than when the obstruction is mechanical; and occasionally, after a few days, give little evidence of deficiency of bile. The urine is deeply tinged. When the disease assumes its more active and febrile form, those symptoms referrible to the brain and nervous system, and which appear to depend upon the deleterious effects of bile circulating in the blood, are more intensely marked than in any other form of jaundice; and the tendency to hæmorrhage sometimes comes on very early, and is excessive. In some cases, rigors, which assume the form of irregular intermittent paroxysms, form a prominent feature, as the disease advances; and then it often happens, though not always, that suppuration is established; and this may be going on to a great extent, while still the jaundice has rather decreased, or varied exceedingly in its intensity." (P. 614.)

The condition of the liver varies according to the period at which the disease proves fatal; in general, it is not enlarged, and not unfrequently diminished in size. No bile is found in the minute ducts, and but little in the gall bladder. When the disease has terminated early, the liver feels rather soft: the surface appears variegated, of a light yellow and dark red or purple in patches, and certain portions project above the rest. In more protracted cases, where the skin is of that light lemon-colour which often bespeaks a very general disorganization of the liver, the structure is extensively altered, the acini whitish and hard, forming groups and clusters, generally forming a sheath around the portal vessels. In other cases the liver is pervaded with numerous abscesses.

"With regard to treatment, in cases where jaundice depends upon inflammatory action, it must always be decidedly antiphlogistic; but it is only where it presents itself under the more violent forms, that general bleeding need be employed. In other cases, cupping from the margin of the ribs, and (as soon as the bleeding is stopped) the assiduous application of poultices over the liver, will be most important remedies. The combination of calomel, antimony, and opium, must be occasionally administered; and antimonials must be combined with the purgatives, which, in the form of pills, should be given to act regularly on the bowels, and should be aided occasionally by the sulphate of magnesia and other saline purgatives: and, in many cases, the saline purgatives, alternated occasionally with mercurials, are sufficient to cure the disease. A free and uninterrupted action from the skin is most desirable; and to promote this more effectually, the warm-bath may be very advantageously employed; and the poultice, while it restrains the patient in bed, assists forcibly, as a diaphoretic measure. Under treatment of this kind, a very large proportion of cases are completely cured; and where the result is otherwise, it generally arises from some complication of diseases; most frequently from previous disorganization of the liver, or from neglect, on the part of the patient." (P. 616.)

Eight cases are reported illustrating this form of jaundice.

Dr. Bright, in the Gulstonian Lectures in 1833, stated his views on the situation and structure of malignant diseases of the liver, and they were published at the time, we believe, in the Medical Gazette. He again brings them forward, and illustrates them with cases and drawings. We must refer our readers to the Reports themselves for the proofs of the point which Dr. Bright wishes to establish, that the chief, if not the only early development of the disease is in the cellular membrane connecting the acini, rather than in the acini themselves. Both the papers of Dr. Bright are illustrated by engravings of much beauty.

The remaining papers are "*a Case of Wound of the Abdomen followed by Protrusion of a large Portion of Omentum*," by Mr. KEY, in which the omentum, which could not be returned without dilating the wound, was allowed to remain, and it gradually shrunk; some "*Ophthalmic Cases*" very judiciously treated, by Mr. MORGAN; and a remarkable "*Case of Misplacement of the Stomach*," by Dr. BRIGHT. This last we regret that we cannot give entire; and it will not admit of abridgment.

